# Pathways for the formation of ice polymorphs from water predicted by a metadynamics method

**DOI:** 10.1038/s41598-020-61773-x

**Published:** 2020-03-13

**Authors:** Hiroki Nada

**Affiliations:** National Institute of Advanced Industrial Science and Technology (AIST), 16-1 Onogawa, Tsukuba, 305-8569 Japan

**Keywords:** Chemistry, Materials science, Mathematics and computing, Physics

## Abstract

The mechanism of how ice crystal form has been extensively studied by many researchers but remains an open question. Molecular dynamics (MD) simulations are a useful tool for investigating the molecular-scale mechanism of crystal formation. However, the timescale of phenomena that can be analyzed by MD simulations is typically restricted to microseconds or less, which is far too short to explore ice crystal formation that occurs in real systems. In this study, a metadynamics (MTD) method was adopted to overcome this timescale limitation of MD simulations. An MD simulation combined with the MTD method, in which two discrete oxygen–oxygen radial distribution functions represented by Gaussian window functions were used as collective variables, successfully reproduced the formation of several different ice crystals when the Gaussian window functions were set at appropriate oxygen–oxygen distances: cubic ice, stacking disordered ice consisting of cubic ice and hexagonal ice, high-pressure ice VII, layered ice with an ice VII structure, and layered ice with an unknown structure. The free-energy landscape generated by the MTD method suggests that the formation of each ice crystal occurred via high-density water with a similar structure to the formed ice crystal. The present method can be used not only to study the mechanism of crystal formation but also to search for new crystals in real systems.

## Introduction

Ice crystals have a variety of polymorphs depending on the temperature and pressure^1,2^. Ice polymorphs play essential roles in the Earth’s environment, the life cycles of organisms living in cold environments, our daily life, and industry. Therefore, numerous studies have investigated ice polymorphs to determine the phase diagram, identify suitable formation conditions, and so forth^1–4^.

However, the formation mechanisms of most ice polymorphs remain elusive. Even for ordinary hexagonal ice I (I_h_), the formation mechanism is still unclear. One of the reasons for this is that appropriate methods for determining the formation mechanisms of ice polymorphs are lacking. To resolve this issue, it is necessary to analyze the molecular-scale processes of the structural changes that occur during the formation of ice polymorphs. Computer simulations, such as molecular dynamics (MD), are often useful for this purpose^5^. However, the formation of each ice polymorph occurs via nucleation, which is an activation process that occurs on a much longer timescale than the typical microsecond-order run time of an MD simulation. Thus, the timescale of MD simulations is far too short to study the molecular-scale processes of the structural changes that occur in the formation of ice polymorphs.

Metadynamics (MTD) is an enhanced sampling method that increases the probability of reaching high free-energy states by adding a Gaussian bias potential to the Hamiltonian of a state^6,7^. The MTD method has been used to analyze reaction pathways^8–15^, the conformations of additives at crystal surfaces^16–19^, crystal structures^20–25^, crystal nucleation^26–33^, drug design^34–38^, and the transport mechanism of an ion in a sub-nanopore^39^. The MTD method affords a free-energy landscape in a space of collective variables (CVs) for a system of interest. This free-energy landscape quantitatively represents the relative thermodynamic stabilities of many different states of the system. Therefore, it is possible to search for a kinetically favorable pathway for the transition between different states, on the assumption that this transition occurs according to a pathway on the free-energy landscape. Thus, application of the MTD method to supercooled water is anticipated to yield valuable information for elucidating the formation mechanisms of ice polymorphs.

Quigley and Rodger adopted the MTD method in an MD simulation of supercooled water using the Steinhardt order parameters and the potential energy, *U*, as CVs^26^. Although the formation of ice I occurred, the formation of other ice polymorphs did not. In principle, the number of states that are reproduced in an MTD simulation increases with an increasing number of CVs. However, the number of CVs should be set as small as possible to conserve computational time and facilitate the determination of pathways for the formation of each polymorph^7^. Moreover, it is preferable to use experimentally measurable CVs to enable experimental verification of the formation mechanism. Recently, Niu *et al*. applied the MTD method to examine the formation mechanism of a silica crystal using X-ray diffraction peak intensities, which can be measured experimentally, as CVs^31^.

In this study, MD simulations in which the MTD method was implemented (MTD simulations) were performed for low-density water (LDW) using only two CVs, which were two discrete oxygen–oxygen radial distribution functions (RDFs)^40^ represented by Gaussian window functions with different intervals of integration. RDFs are measurable experimentally. The obtained results demonstrate that by selecting an appropriate set of intervals of integration, the formation of different ice polymorphs, including an unknown ice structure, can occur in a single MTD simulation. Kinetically favorable pathways for the formation of each observed ice polymorph, which were predicted by the free-energy landscape, are also presented.

## Results and Discussion

### Ice polymorphs formed in MTD simulations

Two discrete RDFs implemented in PLUMED 1.3^40^ were selected as CVs, with each represented by a Gaussian window function, *w*(*r*), as a function of the oxygen–oxygen distance, *r*, so that CVs were differentiable with *r*^40^. Each discrete RDF represented the number of *r* in a given range. Four different sets of CV pairs with different intervals of integration for *w*(*r*) were examined (sets 1–4, see Table 1). *w*(*r*) was set in the short-*r* region for each CV of sets 1 and 2 and in the long-*r* region for each CV of sets 3 and 4. Previous studies indicate that the use of *U* as a CV is convenient for reproducing crystallization in MTD simulations^26,27^. Thus, a CV set of both the RDFs and *U* was also examined (set 5).

**Table 1:** Collective variable (CV) sets examined in this study.

	*W*	CV1(RDF)			CV2(RDF)			CV3(*U*)	*t*_*tot*_
		*a*	*b*	*σcv*	*a*	*b*	*σcv*	*h*	
	(kJ/mol)	(nm)	(nm)	(nm)	(nm)	(nm)	(nm)	(kJ/mol)	(ns)
set 1	0.50	0.25	0.32	0.07	0.30	0.40	0.10		460
set 2	0.25	0.25	0.32	0.07	0.40	0.47	0.07		330
set 3	0.25	0.50	0.60	0.10	0.60	0.70	0.10		315
set 4	0.25	0.60	0.70	0.10	0.70	0.80	0.10		590
set 5	0.25	0.25	0.32	0.07	0.30	0.40	0.10	0.5	400

The CVs of the two discrete RDFs are denoted CV1 and CV2. The CV of *U* is denoted CV3. *W* is the height of the bias potential. *σ*_CV_ is the width of the Gaussian window function with integration from *a* to *b*, $w(r)={(2\pi )}^{-1/2}{N}^{-1}{{\sigma }_{{\rm{CV}}}}^{-1}{\sum }_{i}{\sum }_{j\ne i}{\int }_{a}^{b}\exp (-{2}^{-1}{{\sigma }_{{\rm{CV}}}}^{-2}{(R-{r}_{ij})}^{2})dR$, where *N* is the number of water molecules in CV1 and CV2. *t*_*tot*_ is the total run time of the MTD simulation. *h* is the effective width of the bias potential in CV3^40^. The values of *a* and *b* for CV1 were determined such that the discrete RDF corresponded to the 1^st^ peak region of the RDF for sets 1, 2, and 5, the 2^nd^ minimum region for set 3, and the 3^rd^ peak region for set 4. Those values for CV2 were determined such that the discrete RDF corresponded to the 1^st^ minimum region of the RDF for sets 1 and 5, the 2^nd^ peak region for set 2, the 3^rd^ peak region for set 3, and the 3^rd^ minimum region for set 4.

Interestingly, during the MTD simulations for sets 1–4, not only spatially uniform structures but also spatially nonuniform structures in which high-density areas and low-density (or vacuum) areas coexisted appeared in the system. Notably, the structure in the high-density areas can be regarded as having been formed at high pressure. Thus, in addition to the formation of ice I, the formation of high-pressure ice polymorphs can also be expected in a single MTD simulation with these CV sets.

The formation of ice VII, which is a high-pressure ice polymorph, occurred for sets 1–4. The formation of metastable cubic ice I (I_c_) also occurred for sets 3 and 4. In principle, the probability of the formation of different ice polymorphs in a single MTD simulation increases if *w*(*r*) is set in the long-*r* region for each CV because the difference in the CV value between ice polymorphs increases upon increasing the *r* region at which *w*(*r*) is set (Fig. S1), facilitating distinguishing between different ice polymorphs in the CV space. In this study, the CV values of water, I_h_ (and I_c_), and high-density ice polymorphs at 200 K and 1 atm in the *r* region selected for sets 3 and 4 displayed distinct differences (Fig. S1), indicating that these CV sets were appropriate for the formation of different ice polymorphs in an MTD simulation.

**Figure 1:**
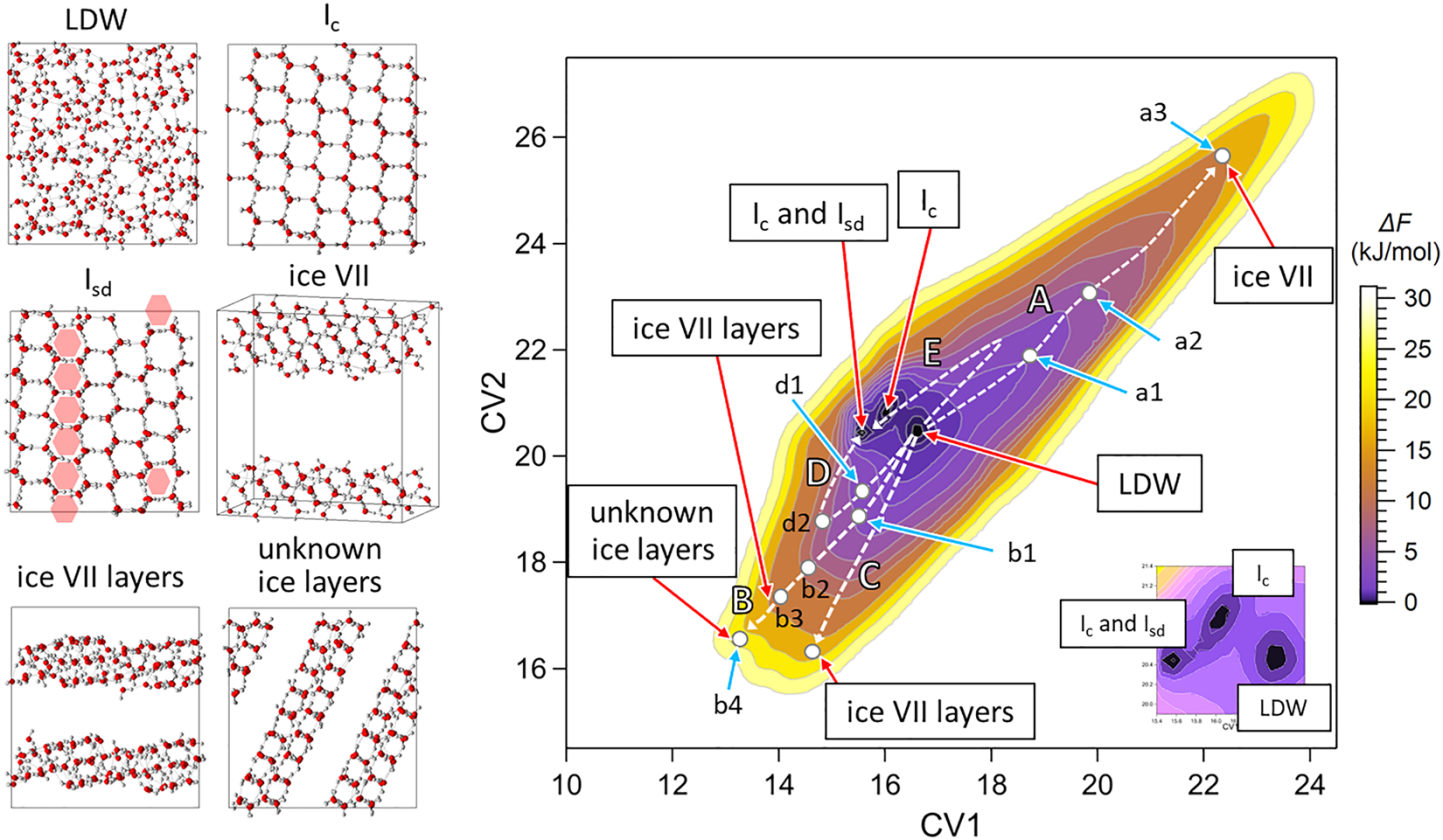
Snapshots of typical structures of low-density water (LDW) and the ice structures in the metadynamics simulation (I_c_, I_sd_, ice VII, ice VII layers, and unknown ice layers) (left) and the free-energy landscape (right) for set 4. I_h_ structures appear in I_sd_ (pink hexagons). Collective variable (CV) regions at which each ice structure or LDW appears (red arrows). Predicted kinetically favorable pathways for the formation of the ice structures (dashed white arrows): A for ice VII, B for ice VII layers and unknown ice layers, C for ice VII layers, and D and E for I_c_ and I_sd_. The free-energy landscape represents the free-energy difference, *∆F*, at each state from the absolute minimum at (CV1, CV2) = (15.6, 20.5).

However, ice VII did not appear for set 5, although I_c_ was observed. One reason for this could be that the simulation run of 400 ns was still too short for the system to reach high-energy states including high-pressure ice polymorphs in the CV space represented by three CVs, which created a substantially larger number of different states than in the case of two CVs. In the remainder of this paper, we will focus on set 4. The MTD simulation for this set indicated the formation of not only I_c_ and ice VII but also I_c_ in which the structure of I_h_ was partially included (stacking disordered ice, I_sd_^41^), layered ice VII (ice VII layers), and layered ice with an unknown structure (unknown ice layers) (Fig. 1). Earlier MD simulation studies have also reported the formation of unknown ice, which had a different structure from the unknown ice layers formed in the present simulations^42,43^. To my knowledge, this study is the first to detect an unknown ice structure using the MTD method. Results of the MTD simulations for sets 1–3 and 5 are presented in Figs. S2–S5.

### Pathways for the formation of each ice phase

The free-energy landscape obtained for set 4 is presented in Fig. 1. Suppose that the transition between different phases preferentially occurs along a pathway for which the gradient of the free-energy landscape is gentle. Then, a kinetically favorable pathway for the formation of ice VII from LDW is expected (A in Fig. 1). Similarly, the pathways for the formation of ice VII layers and unknown ice layers are expected (B and C, and B, respectively, in Fig. 1). Although the ice VII layers and unknown ice layers were artifacts of the present MTD simulation, their appearance suggests that the formation of the ice structures constituting those ice layers is kinetically favorable.

**Figure 2:**
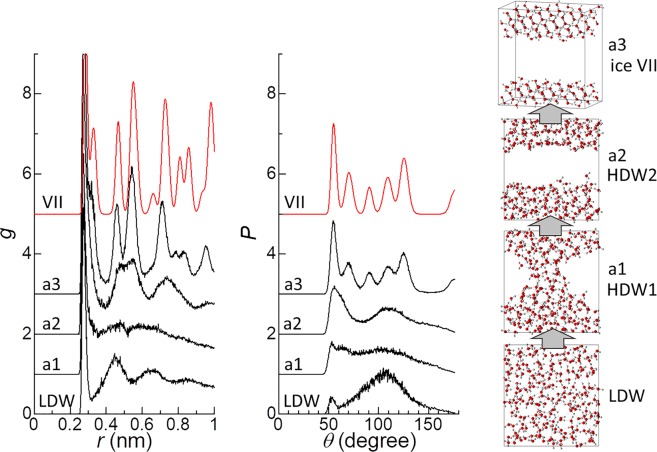
Oxygen–oxygen pair distribution function (*g*), the distribution function of the angle, *θ*, formed by the three nearest-neighbor oxygen atoms (*P*), and a snapshot of typical structures of low-density water and three states along pathway A (a1, a2, and a3) on the free-energy landscape (Fig. 1). *g* and *P* for bulk VII are presented for comparison, obtained by performing a molecular dynamics simulation of bulk (1024 H_2_O) ice VII at 200 K and 2 GPa.

**Figure 3:**
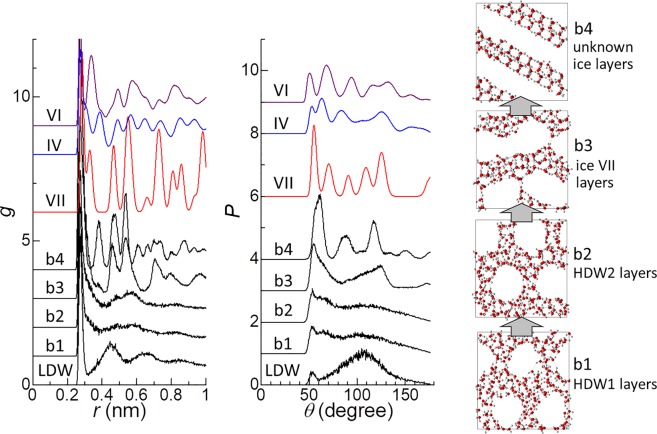
Plots of *g* and *P*, and snapshot of typical structures of four states along pathway B (b1, b2, b3, and b4) on the free-energy landscape (Fig. 1). For comparison, *g* and *P* for bulk ice VII, bulk ice IV, and bulk ice VI are also shown. *g* and *P* for bulk ice IV and bulk ice VI were obtained by performing molecular dynamics simulations of ice IV (1536 H_2_O) and ice VI (640 H_2_O) at 200 K and 1 GPa.

The presence of structures at points a1 and a2 along pathway A in the CV space (Fig. 1) suggest that the formation of ice VII occurs via high-density water (HDW) phases (Fig. 2). The oxygen–oxygen pair distribution function (*g*) and the distribution function of the angle, *θ*, formed by the three nearest-neighbor oxygen atoms (*P*) at a1 and a2, suggest that the HDW structures at these points were different; the HDW at a1 (HDW1) had a disordered structure, whereas that at a2 (HDW2) had an ordered structure resembling the structure of ice VII (Fig. 2). Similarly, the formation of ice VII layers and unknown ice layers along pathway B was found to occur via HDW layers with a disordered structure (HDW1 layers) and layers with an ordered structure resembling the structure of ice VII (HDW2 layers) (Fig. 3).

The formation of the unknown ice layers from the ice VII layers suggests that the unknown ice layers had a structure that resembles ice VII. However, it is speculated that the structure of the unknown ice layers also resembled that of ice IV or ice VI. A plot of *g* for the unknown ice layers displayed a distinct peak at *r* = 0.38 nm (b4), where the first minimum of *g* appears for most of the other ice polymorphs (Fig. 3). An ice polymorph that produces a peak around *r* = 0.38 nm is ice IV, which is known to appear as a metastable phase within the stability field of ice V^44^. Ice VI also produces a peak in the region of the first minimum for most of the other ice polymorphs, although it appears at the smaller *r* value of 0.34 nm. At present, it is unclear whether the formation of the unknown ice layers is related to the formation of ice IV or ice VI in real systems. There is the possibility that this formation corresponds to the discovery of a new ice structure in real systems. More detailed studies of the formation of the unknown ice layers, including first-principles calculations and MD simulations using different potential models, should be performed in the future. Snapshots of the structure of the unknown ice layers viewed from different orientations with information about an expected unit cell structure are presented in Fig. S6.

**Figure 4:**
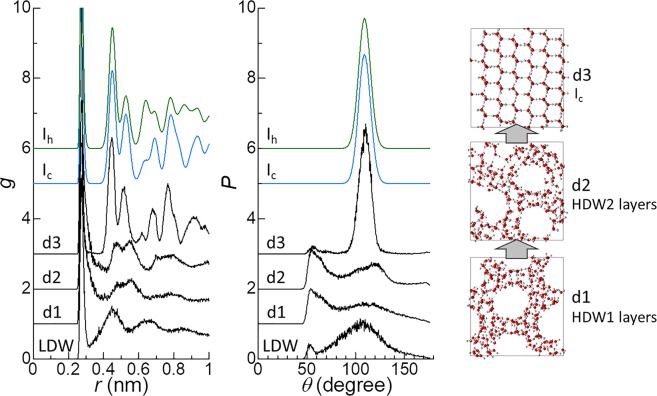
Plots of *g* and *P* and snapshots of typical structures of three states along pathway D (d1, d2, and d3) on the free-energy landscape shown in Fig. 1. For comparison, *g* and *P* for bulk I_c_ and I_h_ are also shown. *g* and *P* for bulk I_c_ and I_h_ were obtained by performing MD simulations of I_c_ (512 H_2_O) and I_h_ (2880 H_2_O) at 200 K and 1 atm.

It was difficult to determine the pathway for the formation of I_c_ and I_sd_ from only the free-energy landscape, because the CV region at which their formation occurred was close to that of LDW. During the simulations, the formation of I_c_ and I_sd_ frequently occurred via HDW1 and HDW2 or HDW1 layers and HDW2 layers, in addition to their formation directly from LDW. Thus, D and E shown in Fig. 1 are also considered to be the pathways for the formation of I_c_ and I_sd_. An example of the formation of I_c_ along pathway D observed in the simulations is shown in Fig. S7. Similar to the cases of the formation of ice VII and ice VII layers, the formation of I_c_ along pathway D or E occurred via HDW1 and HDW2 or HDW1 layers and HDW2 layers (Fig. 4). A plot of *g* at d2 reveals the structure of the HDW2 layers, which were formed prior to the emergence of I_c_, resembling that of I_c_ (Fig. 4).

The free-energy landscape shown in Fig. 1 revealed only three local minima, one of which corresponded to LDW and the others to I_c_ (and I_sd_) (see the magnification of the free-energy landscape shown in the lower right region in Fig. 1). As the ideal structure of I_c_ did not fit the present simulation system, the formation of distorted I_c_ with various orientations occurred, which resulted in the division of the CV region for I_c_ into two adjacent local minima. Strictly speaking, the accuracy of the free energy at each state shown in the free-energy landscape was not enough high to confirm the number of local minima and their relative depths, because the height of the bias potential (0.25 kJ/mol) was not sufficiently small. Similarly, the accuracy of the gradients of the free-energy landscape near the local minima were also not enough high to judge whether the gradient along a pathway for the formation of I_c_ (and I_sd_) directly from LDW was gentle. The MTD simulation with the well-tempered MTD method^45^ is needed to determine the number of local minima, their relative depths, and the gradient of the free-energy landscape near the local minima precisely.

It is obvious that the thermodynamic stabilities of ice VII, ice VII layers, and unknown ice layers, which appeared only at high free-energy regions, were much lower than those of LDW, I_c_, and I_sd_. The frequent formation of these high-energy ice structures in spite of their low thermodynamic stabilities suggests that their formation was kinetically favorable compared to the formation of other structures in the corresponding CV regions.

In the present simulations, the formation of other ice polymorphs did not occur. A possible reason for it is that the formation of water with the same CV values as other ice polymorphs prevented the formation of them, which can be speculated from the result that the CV values for ice IV and VI were close to the values for HDW (Fig. S1). Systematic MTD simulations with different CV sets should be done to confirm this speculation.

### Comparison with real systems

In real systems, ice VII appears not only in the region where it is thermodynamically stable on the phase diagram but also in the region where ice VI or ice VIII is thermodynamically stable^46,47^, suggesting that the formation of ice VII is kinetically more favorable than the formation of ice VI or ice VIII. The structure of water at high pressure is known to be closer to that of ice VII than to that of ice VI^47,48^, which explains the kinetically favorable formation of ice VII in the stable region for ice VI. These findings are qualitatively consistent with the present result that ice VII appeared from HDW2, which had a similar structure to ice VII, although the simulation temperature of 200 K was considerably lower than the experimental temperature at which the formation of ice VII has been observed in the stable region of ice VI or ice VIII (room temperature or higher).

In the present MTD simulation, I_c_ and I_sd_ appeared but I_h_ did not. This result suggests that the formation of I_c_ was kinetically more favorable than that of I_h_, which is consistent with the experimental finding of Takahashi and Kobayashi that, at temperatures much lower than the melting point of I_h_, the formation of I_c_ is kinetically more favorable than the formation of I_h_^49^. It should be further noted that the appearance of I_c_ and I_sd_ rather than I_h_ was also reported in an MTD simulation study by Quigley and Rodger^26^, an MD simulation study by Moore and Molinero^50^, and an MD simulation study by Malkin *et al*^51^.

Thus, the present MTD simulation results were qualitatively consistent with previous MD and MTD experimental studies. To confirm that the results are not dependent on the system size, the MTD simulations were also performed for a large system containing approximately four times as many water molecules (896 H_2_O) as the aforementioned system (216 H_2_O), using almost the same CVs as for set 4. The formation of both I_c_ and ice VII occurred even in the MTD simulations of the large system (Fig. S8). MTD simulations using different potential models remain to be investigated in a future study.

## Conclusions

MTD simulations of supercooled water using two discrete RDFs, represented by *w*(*r*) with different intervals of integration as CVs, successfully reproduced the formation of various ice structures when the interval of integration for the *w*(*r*) of each CV was set appropriately: I_c_, I_sd_, ice VII, ice VII layers, and unknown ice layers. To our knowledge, the present study is the first to report the formation of both I_c_ (or I_sd_) and high-pressure ice polymorphs in a single MTD simulation.

The free-energy landscape generated via the present MTD method indicated that LDW, I_c_, and I_sd_ corresponded to local minimum structures, whereas the formation of the high-energy structures of ice VII, ice VII layers, and unknown ice layers occurred because they were kinetically favorable. The present results indicate that the formation of ice VII from LDW occurred via HDW2, which has an ordered structure similar to that of ice VII. The present results also suggest that the formation of I_c_ and I_sd_ was kinetically more favorable than that of I_h_. These findings of the present MTD simulation are consistent with earlier MTD, MD, and experimental studies^46–51^.

In conclusion, MTD simulations using CVs of discrete RDFs show great potential for elucidating the formation mechanisms of ice polymorphs in real systems. The present MTD simulations have provided valuable information on the formation mechanisms of I_c_ (and I_sd_) and ice VII and also point toward the possibility of discovering a new ice structure. The MTD simulations with the present CVs generated not only spatially uniform structures but also nonuniform structures in the system, so it is anticipated that the present method can be used to analyze not only the thermodynamic stability and formation mechanism of bulk phases but also those of phase separation processes, such as separation into high- and low-density water phases or amorphous phases^52,53^. The MTD method using appropriately selected CVs therefore represents a new strategy for studying crystal growth physics, phase transition physics, materials science, and materials technology.

### Simulation methods

The simulations were performed for a constant-volume rectangular parallelepiped system consisting of 216 water molecules. The initial structure of the system was obtained as follows. Initially, the system in which all water molecules were arranged into the lattice sites of bulk I_h_ consisting of 3 × 3 × 3 unit cells, each of which had an antiferroelectric structure containing eight water molecules^54^, was heated by an MD simulation at 800 K for 1 ns. Subsequently, the system was cooled by an MD simulation at 200 K and 1 atm for 20 ns. The final structure obtained after the cooling MD simulation was used as the initial structure of the system. The dimensions of the system were 1.367 × 2.277 × 2.137 nm^3^. The constant-pressure algorithm was not used with the present MTD method because it might cause large changes in the volume of the system^55^, which would create a substantially large number of different states including vapor states.

A modified version of the six-site model of H_2_O^56^, which was proposed for the simulation of ice crystal growth from water near the real melting point^57^, was used to estimate the intermolecular interactions. The long-range Coulomb interaction was calculated using the Ewald method with a convergence parameter of 5.0333 nm^−1^, 7 × 11 × 11 reciprocal lattice vectors, and a real-space cut-off distance of 0.65 nm. The short-range Lennard-Jones interaction was cut off at an intermolecular distance of 0.65 nm.

The computations were performed using the leapfrog algorithm with a time step of 2 fs. The temperature was maintained at 200 K using the Nosé–Hoover thermostat with a coupling constant of 0.1 ps^58^. The MD simulations were performed using DL_POLY 2.20^59^ in which PLUMED 1.3^40^ was implemented to permit combination with the MTD method. For the analysis of *P*, the oxygen atoms in a selected oxygen atom pair was judged to be nearest neighbors to each other if the interatomic distance was shorter than 0.3 nm.

## Supplementary information

Supplementary Information.
