# Supplementary material for: Pathways for the formation of ice polymorphs from water predicted by a metadynamics method
Source: Sci Rep. 2020 Mar 13;10:4708. doi: 10.1038/s41598-020-61773-x (PMC7069948; doi:10.1038/s41598-020-61773-x)
Supplement: Supplementary file 1 — Supplementary Information. [file 41598_2020_61773_MOESM1_ESM.pdf]

Supplementary Information

Pathways for the formation of ice polymorphs from water predicted by a metadynamics method

Hiroki Nada*

National Institute of Advanced Industrial Science and Technology (AIST), 16-1 Onogawa, Tsukuba 305-8569, Japan.

*email: hiroki.nada@aist.go.jp

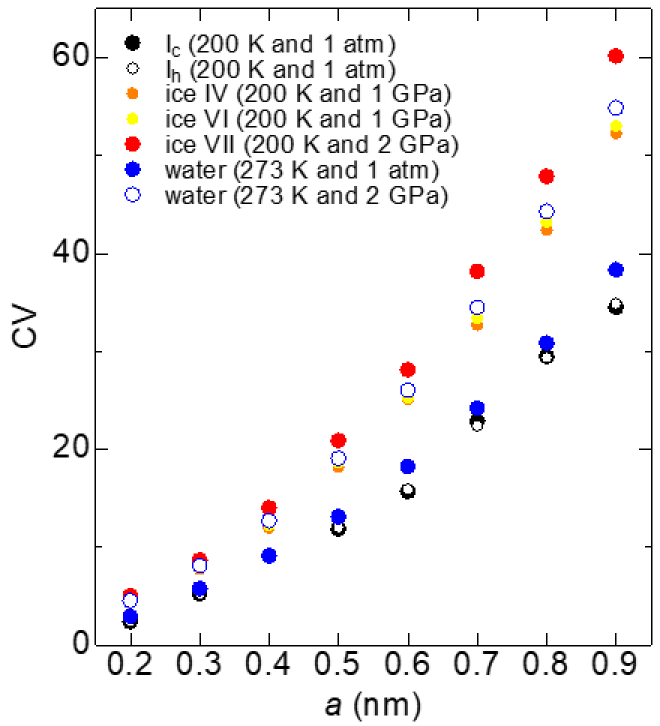

Figure S1. Collective variable (CV) values for high-density water (HDW), low-density water (LDW), and several different ice polymorphs, which were calculated using equation $w(r) = (2\pi)^{-1/2} N^{-1} {\sigma_{\rm CV}}^{-1} \sum_i \sum_{j \neq i} \int_a^b \exp\left(-2^{-1} {\sigma_{\rm CV}}^{-2} \left(R - r_{ij}\right)^2\right) dR$, where $\sigma_{\rm CV}$ (=0.1 nm) is the width of the Gaussian window function with integration from *a* (nm) to *b* (=*a*+0.1) (nm) and *N* is the number of water molecules. It can be seen that the relative relationship of CV between HDW, LDW, and the ice polymorphs does not significantly change even if *a* is slightly changed Thus, it is natural to assume that slight changes of *a* and *b* do not significantly change the simulation results.

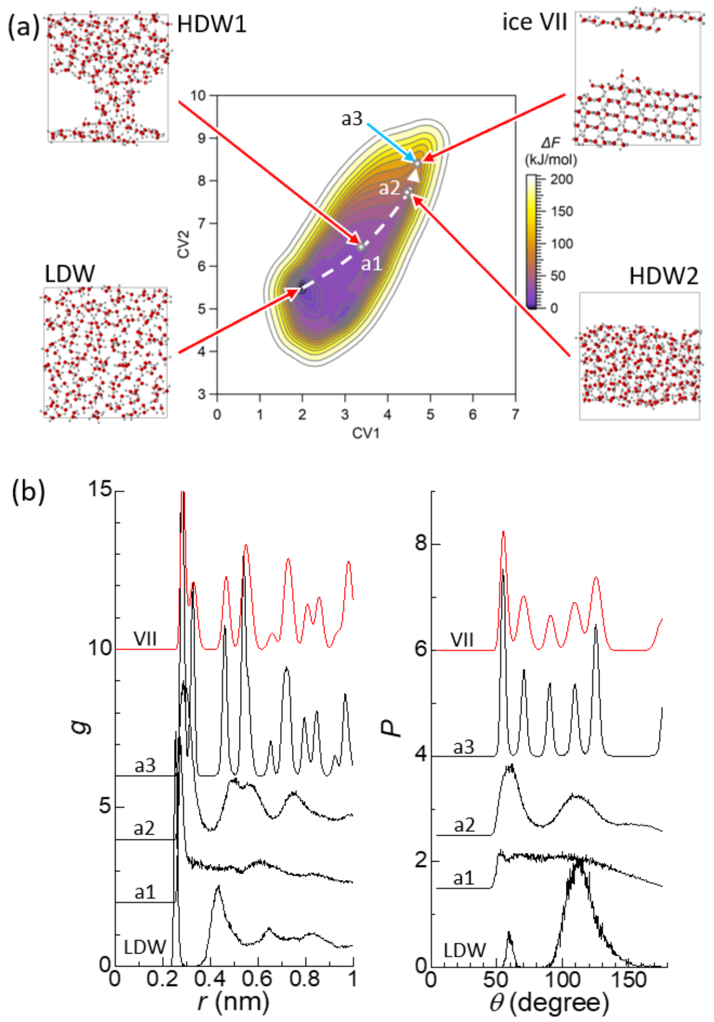

Figure S2. (a) The free-energy landscape and snapshots of typical structures of low-density water (LDW), high-density water (HDW) with a disordered structure (HDW1), HDW with an ordered structure (HDW2), and ice VII along pathway, A, for the formation of ice VII in the metadynamics (MTD) simulation for set 1. (b) Oxygen–oxygen pair distribution function (*g*) and the distribution function of the angle, *θ*, formed by the three nearest neighbor oxygen atoms (*P*) along pathway A (a1, a2, and a3) on the free-energy landscape. *g* and *P* for bulk VII are presented for comparison, obtained by performing a molecular dynamics (MD) simulation of bulk (1024 H_2O) ice VII at 200 K and 2 GPa.

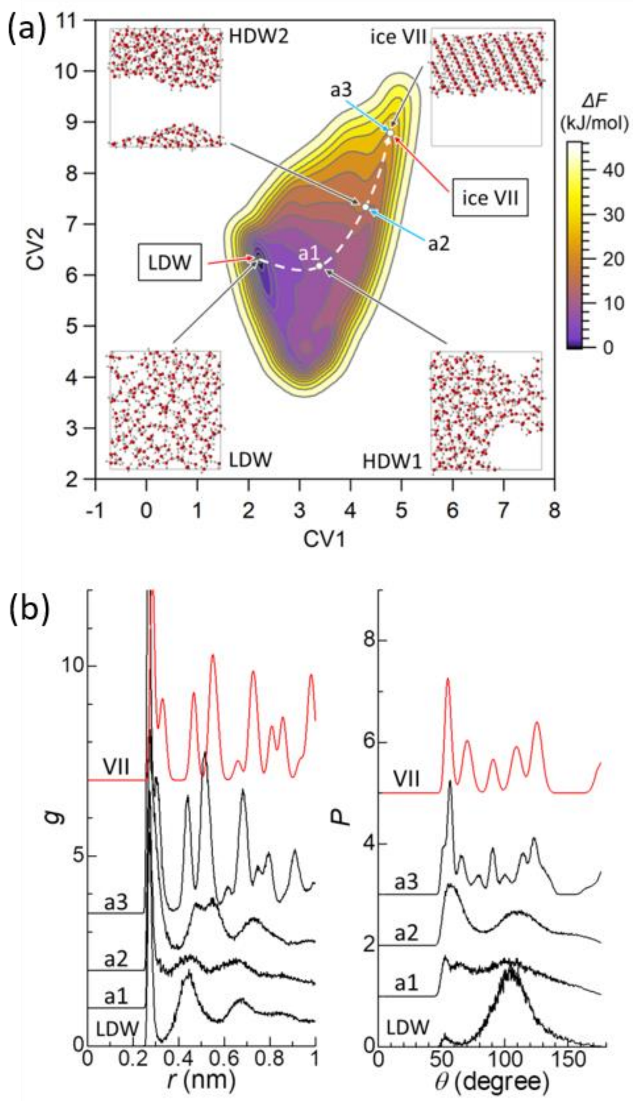

Figure S3. (a) The free-energy landscape and snapshots of typical structures of LDW, HDW1, HDW2, and ice VII along pathway, A, for the formation of ice VII in the MTD simulation for set 2. (b) *g* and *P* along pathway A (a1, a2, and a3) on the free-energy landscape.

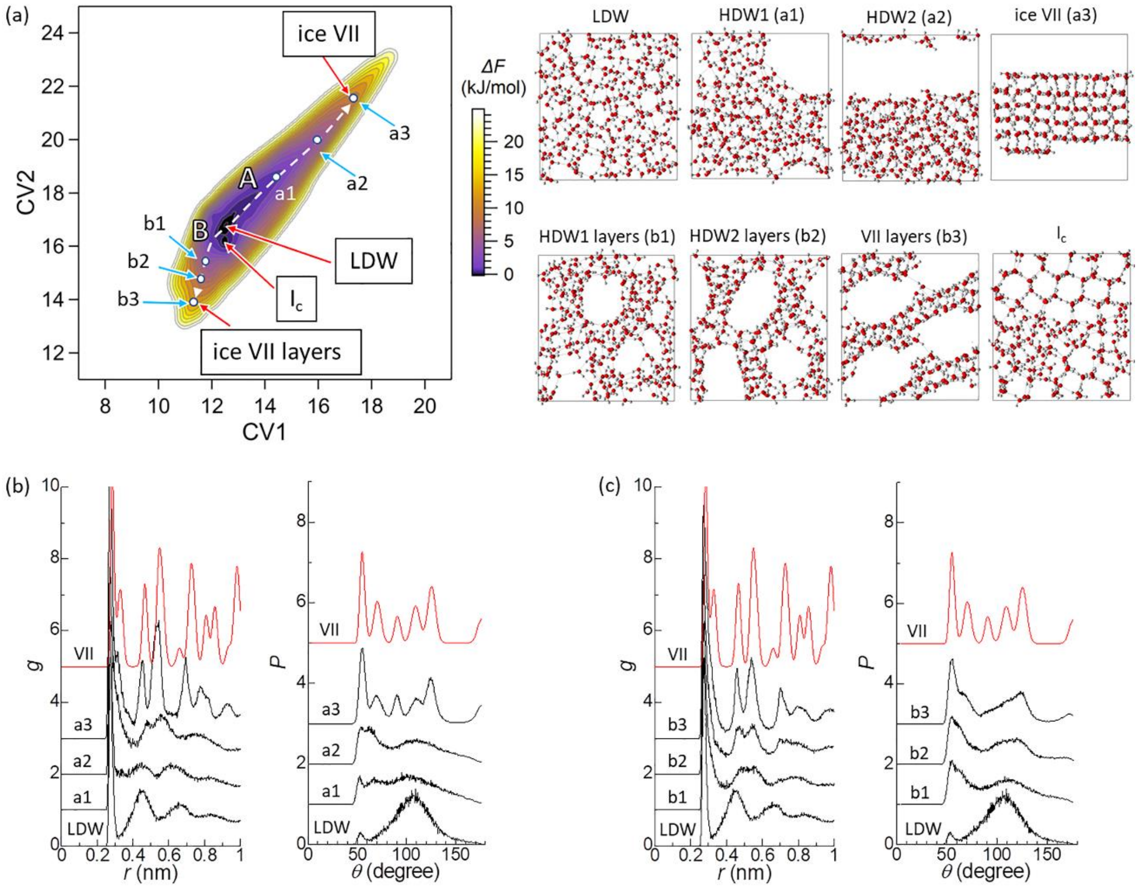

Figure S4. (a) The free-energy landscape, snapshots of typical structures of LDW, HDW1, HDW2, and ice VII along pathway, A, for the formation of ice VII, snapshots of typical structures of HDW1 layers, HDW2 layers, and ice VII layers along pathway, B, for the formation of ice VII layers, and a snapshot of typical structure of I_c in the MTD simulation for set 3. (b) *g* and *P* along pathway A (a1, a2, and a3) on the free-energy landscape. (c) *g* and *P* along pathway B (b1, b2, and b3) on the free-energy landscape.

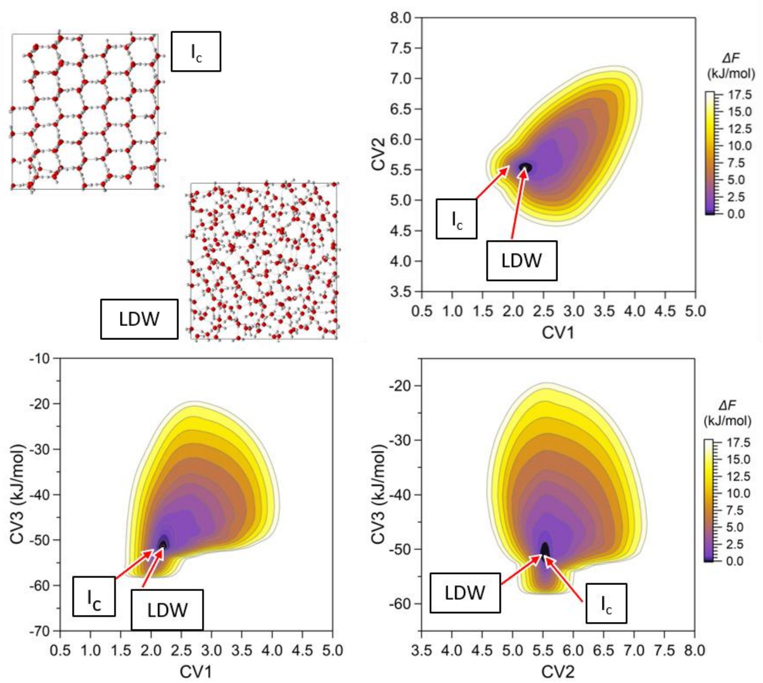

Figure S5. Snapshots of typical structures of LDW and I_c and the free-energy landscapes in the MTD simulation for set 4.

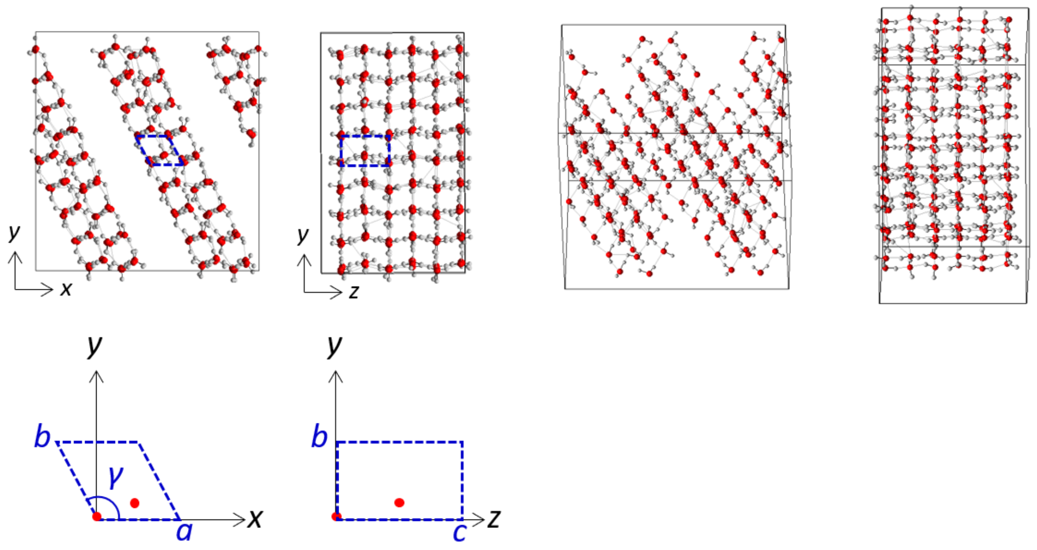

Figure S6. Structure of the unknown ice layers viewed from four different directions. The unit cell is expected to be a monoclinic system with two water molecules (see the region enclosed by dashed blue lines); a = 0.304 nm, b = 0.284 nm, c = 0.456 nm, $\alpha = \beta = 90°$, and γ = 117°. The Cartesian coordinates of water molecules in the unit cell (red circles) are (x [nm], y [nm], z [nm]) = (0.0, 0.0, 0.0) and (0.152, 0.074, 0.228).

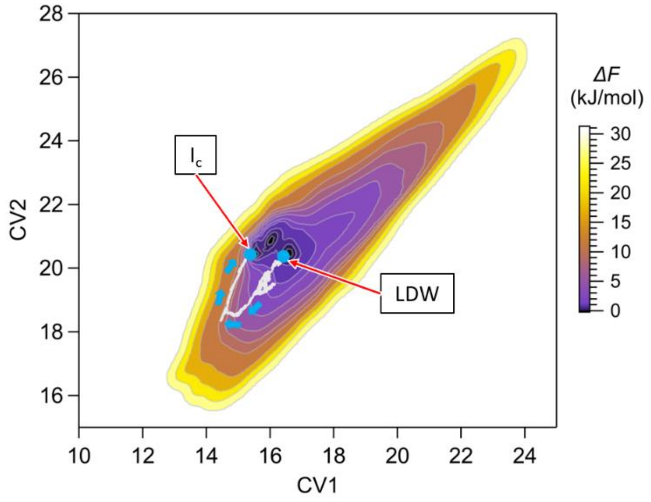

Figure S7. An example of the time-sequence of the CV values during the formation of I_c from LDW observed in the simulations (see the trajectory drawn by white lines).

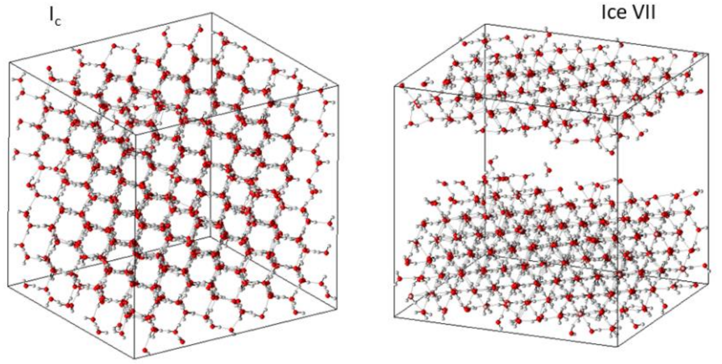

Figure S8. Typical structures of I_c and ice VII formed in the metadynamics (MTD) simulations of the large system containing 896 H_2O molecules at 200 K. The method used for the MTD simulations was the same as that described in the text. The formation of I_c occurred during a 120-ns MTD simulation with set 4. The formation of ice VII occurred during an 80-ns MTD simulation with set 4 in which the height of the bias potential *W* was increased to 5.0 kJ/mol, such that the system reached high-energy states within the short run time. Thus, it is expected that the formation of both I_c and ice VII would occur in a single MTD simulation with set 4 if the simulation run were substantially longer than 120 ns.
